# Toward Personalized Anticoagulation: Clinical Predictors of Early Warfarin Response in Heart Valve Replacement Patients

**DOI:** 10.3390/biomedicines14020446

**Published:** 2026-02-17

**Authors:** Rania Abdel-latif, Shaban Mohammed, Tamer Abdalghafoor, Rana Mekkawi, Cornelia Sonia Carr, Abdulaziz M. Alkhulaifi, Ali Kindawi, Mohd Lateef Wani, Samim Azizi, Mohamad El-Kahlout, Sankar Balasubramanian, Hatem Sarhan, Samy Hanoura, Sameh Aboulnaga, Yasser Shouman, Abdulwahid Al Mulla, Radja Badji, Wadha Al-Muftah, Amr Salah Omar

**Affiliations:** 1 Qatar Genome Program, Qatar Precision Health Institute, Qatar Foundation, Doha 5825, Qatar; rbadji@qf.org.qa (R.B.); walmuftah@qf.org.qa (W.A.-M.); 2 Department of Pharmacology and Toxicology, Faculty of Pharmacy, Minia University, Minia 61511, Egypt; 3 Pharmacy Department, Hamad Medical Corporation, Doha 3050, Qatar; smohammed3@hamad.qa (S.M.); rm1507467@qu.edu.qa (R.M.); 4 Harefield Hospital, London UB9 6JH, UK; tamer.abdalghafoor@nhs.net; 5 Department of Thoracic and Cardiovascular Surgery, Heart Hospital, Hamad Medical Corporation, Doha 3050, Qatar; ccarr@hamad.qa (C.S.C.); abdulalkh@hamad.qa (A.M.A.); akindawi@hamad.qa (A.K.); mwani@hamad.qa (M.L.W.); sazizi@hamad.qa (S.A.); melkahlout@hamad.qa (M.E.-K.); sbalasubramanian@hamad.qa (S.B.); shanoura@hamad.qa (S.H.); saboulnaga@hamad.qa (S.A.); yshouman@hamad.qa (Y.S.); aalmulla@hamad.qa (A.A.M.); aelsid@hamad.qa (A.S.O.); 6 College of Medicine, Qatar University, Doha 2713, Qatar; 7 Department of Thoracic and Cardiovascular Surgery, Cleveland Clinic, Cleveland, OH 44106, USA; sarhanh@ccf.org

**Keywords:** warfarin sensitivity, mechanical heart valve replacement, anticoagulation management, international normalization ratio, dose prediction model

## Abstract

**Background/Objective**: Warfarin is the standard anticoagulant for patients with mechanical heart valve replacement (HVR). However, its narrow therapeutic index and interpatient variability complicate early postoperative management. Evidence on how valve position influences warfarin sensitivity is limited. This study evaluated the impact of prosthetic valve position and clinical factors on early warfarin response and developed a prediction model to guide initial warfarin dosing in HVR patients. **Methods**: A retrospective study was conducted on 310 adults who underwent mechanical aortic, mitral, or double valve replacement at Hamad Medical Corporation (2015–2022). Warfarin was initiated within 24 h postoperatively, and patients were monitored for three days. Outcomes included daily warfarin dose, international normalized ratio (INR) levels, attainment of therapeutic INR, INR overshoot (≥4), and the warfarin dose index on day 3 (WDI3). Predictors of WDI3 were analyzed using multivariable regression, and a LASSO model was applied to a dose prediction algorithm for the day 1 dose. **Results**: Mitral valve recipients required lower doses than aortic or double valve groups (*p* = 0.008) but had higher INR overshoot rates (18.75% vs. 16.05% and 4.55%; *p* = 0.033). Female sex and a higher baseline INR were associated with greater sensitivity (*p* < 0.01), whereas mitral/double valve position predicted reduced sensitivity (*p* = 0.010). Only half of the cohort reached therapeutic INR by day 3. The prediction model explained ~28% of dose variance with moderate performance. **Conclusions**: Valve position, sex, and baseline INR significantly influence early postoperative warfarin response. Incorporating these clinical factors into dosing algorithms may optimize initial warfarin management in HVR patients.

## 1. Introduction

Valvular heart disease (VHD) is an increasingly prevalent cardiovascular disorder, affecting over 100 million individuals worldwide [1]. Commonly arising from degenerative valve disease and rheumatic fever, VHD promotes hemodynamic disturbances that compromise cardiac function and patient quality of life [2,3]. Surgical valve replacement remains a cornerstone in the management of advanced VHD. Mechanical heart valves offer superior durability compared with bioprosthetic valves; hence, they are recommended in younger patients. However, their heightened thrombogenic potential necessitates lifelong anticoagulation with warfarin [4,5].

Immediate initiation of warfarin therapy after mechanical heart valve replacement (HVR) is standard practice to minimize the risk of thromboembolic events, particularly during the early postoperative phase [6]. Warfarin’s narrow therapeutic index and its highly variable dose–response profile present significant challenges, particularly during the initial phase of treatment, when patients are at high risk for both thrombotic and hemorrhagic events [7]. Patients following HVR are at sustained risk of stroke and valve thrombosis, with this risk varying by valve position. Annual thromboembolic rates are approximately 0.5% for mechanical aortic valves, 0.9% for mitral valves, and up to 1.2% for patients with double valve replacements [8,9]. A thromboembolism rate of 48% per patient per year within the first 10 days following mitral valve replacement (MVR) surgery has been previously reported, highlighting the influence of valve location on thromboembolic risk and complicating the precision of anticoagulant therapy [10].

The therapeutic efficacy of warfarin is monitored by the INR level, with target ranges adjusted according to valve position, typically 2.0 to 3.0 for aortic valves and 2.5 to 3.5 for mitral valves [9]. Postoperatively, patients frequently exhibit heightened sensitivity to warfarin and variability in warfarin response, leading to exaggerated INR responses [11]. Despite this recognized phenomenon, standardized protocols for warfarin initiation in this population are lacking, and empirical initiating dosing—usually starting at 5 mg—remains common practice in many institutions [4,12]. Several clinical factors have been implicated in modulating warfarin sensitivity, including baseline INR, serum albumin, body weight, and co-administration of interacting medications [7,13]. However, the influence of dynamic postoperative changes on warfarin response remains poorly understood [14,15]. Among the less explored determinants is the role of prosthetic valve position. Emerging observations suggest that patients with MVR may exhibit increased sensitivity to warfarin compared with those with aortic valve replacements (AVR), potentially due to differing hemodynamic conditions [16,17,18].

Understanding the impact of valve position and other clinical factors on anticoagulation response is essential for optimizing warfarin dosing strategies and minimizing the risk of adverse outcomes in patients undergoing HVR surgery. Despite the performance of approximately 280,000 valve replacement procedures globally each year, the literature offers limited evidence to guide anticoagulation initiation post-surgery, particularly with respect to prosthesis location. Given these gaps, we hypothesized that valve position—in either the mitral or aortic position—significantly affects warfarin sensitivity and INR level, thereby influencing postoperative anticoagulation management. This study aims to characterize the early postoperative warfarin response based on prosthetic valve position, with the goal of informing a warfarin individualized dosing strategy.

## 2. Materials and Methods

### 2.1. Study Design and Participants

This is a retrospective, single-center, observational study evaluating early postoperative warfarin initiation in adult patients undergoing mechanical cardiac valve replacement, with analyses restricted to the first three days of therapy to assess initial dosing practices and early INR response. Adult patients who underwent mechanical prosthetic heart valve replacement at Hamad Medical Corporation Heart Hospital between February 2015 and November 2022 were included in this study. We screened patient records to identify patients who received mechanical HVR of an aortic, mitral valve, or both, and were initiated immediately on warfarin therapy postoperatively and had their INR monitored for a minimum of 3 consecutive days after warfarin initiation. Patients with bioprosthetic valve replacements, valve-in-valve procedures, and balloon valvuloplasty were excluded. Additionally, patients with significant hepatic dysfunction or those receiving medications that significantly interfere with warfarin metabolism (including amiodarone, rifampin, and antiepileptics) were excluded as well.

The warfarin dose was prescribed in milligrams empirically on a daily basis by the treating physician and modified daily in response to the INR measurements. For all patients, warfarin was initiated 24 h after the surgery, combined with enoxaparin as a bridging agent. Enoxaparin was discontinued once the INR reached the therapeutic target, and warfarin alone was continued. Following the surgery, all patients were monitored in the intensive care unit of HMC Heart Hospital as per the hospital protocol for postoperative care, fluid administration, and extubation. The data collected included patients’ demographics, comorbidities, valve position, postoperative lab measures, and concomitant medications. Additionally, we assessed dates and doses of warfarin in the first 3 days postoperative, the results of the baseline INR, and the INR assessment at least 16 h following warfarin administrations. The therapeutic INR was defined uniformly across the cohort, ranging from 2.0 to less than 4.0. This range has accepted clinical tolerance and was employed to accommodate routine INR fluctuations while maintaining effective anticoagulation for both AVR and MVR patients [9]. INR measurements of greater than 4 were identified as INR overshoots [19]. The warfarin dose index on day 3 (WDI3) was used as a marker for warfarin sensitivity and calculated by dividing the INR on the 3rd day by the mean total warfarin dose [20].

### 2.2. Data Analysis

Descriptive statistics were used to summarize baseline characteristics. Continuous variables were expressed as means ± standard deviations (SDs) or as means and interquartile ranges (IQR: 25–75th percentiles), while categorical variables were displayed as frequencies (%). Group comparisons were performed using Pearson chi-square tests to assess associations between categorical variables. A two-sided *p*-value < 0.05 was considered statistically significant.

To evaluate independent predictors of the warfarin sensitivity index, a multivariate linear regression model was constructed. For the prediction of the day 1 warfarin dose, we used the Least Absolute Shrinkage and Selection Operator (LASSO) regression model. Selection was guided by the Extended Bayesian Information Criterion (EBIC)-based variable selection, followed by post-selection OLS regression for unbiased coefficient estimates. Model performance was assessed using 10-fold cross-validation, with the evaluation based on using the cross-validated R^2^ and root-mean-squared prediction error (RMSE). The correlation between predicted and observed doses was examined using Pearson’s correlation coefficient (r) and the coefficient of determination (R^2^), supported by visual assessment in a scatterplot. A *p* of <0.05 was considered statistically significant. All statistical analyses were performed using Stata version 18.5.

## 3. Results

### 3.1. Baseline Characteristics

A total of 310 patients were included in the analysis, stratified into three groups: aortic valve (n = 164), mitral valve (n = 80), and double valve replacement (n = 66). Female gender was significantly more prevalent in the mitral group (38.75%) compared with the aortic (16.46%) and double valve groups (28.78%) (*p* = 0.001). Heart failure was also more common in the mitral (37.5%) and double valve (33.33%) groups than in the aortic group (18.29%) (*p* = 0.002). Albumin levels showed a borderline difference (*p* = 0.05), with the lowest mean in the mitral valve group. AST levels were significantly higher in the mitral and double valve groups (*p* = 0.047), while ALT levels were comparable across groups (*p* = 0.73) (Table 1).

The mean dose of warfarin administered over the first three postoperative days was significantly different among the study groups. Patients with MVR received the lowest total dose (3.90 ± 1.18; *p* = 0.008). The baseline INR of all patient groups was comparable. However, the INR levels significantly varied among patient groups on day 2 (*p* = 0.013) and day 3 (*p* = 0.022). On day 3, the proportion of patients achieving the therapeutic INR was highest in the aortic group (55.48%), followed by the double (50%) and mitral (41.25%) groups, although this difference was not statistically significant (*p* = 0.06). However, a significantly higher proportion of patients in the mitral group (18.75%) and aortic group (15.85%) had INR overshoot (INR ≥ 4.0) compared with the double valve group (4.54%) (*p* = 0.033). The WDI on day 3 was significantly different among groups; mitral valve recipients showed a tendency toward higher warfarin sensitivity (Table 2).

### 3.2. Determinants of Warfarin Dose Index Following Valve Replacement

A multivariate linear regression analysis was performed to identify factors independently associated with the WDI3 as a marker of warfarin sensitivity.

Patients with mitral or double valve replacement had a significantly lower warfarin sensitivity index compared with those with aortic valves (β = −0.023; *p* = 0.010; 95% CI: −0.041 to −0.005). Female patients showed significantly higher warfarin sensitivity compared with males (β = 0.0502; *p* < 0.001; 95% CI: 0.0373144 to 0.1058574). Similarly, the baseline INR was positively associated with the warfarin sensitivity index (β = 0.141; *p* = 0.007; 95% CI: 0.039547 to 0.2438). Heart failure status, albumin, and AST levels were not significantly associated with the WDI3 (Table 3).

### 3.3. Impact of Valve Position on Empirical Warfarin Dosing Decisions and Predicted Probability of Achieving Therapeutic INR

There was a statistically significant association between valve position and the initial warfarin dose prescribed (*p* = 0.028). Mitral valve recipients were more likely to be prescribed low doses, whereas aortic and double valve recipients more commonly received moderate or high doses (Table 4).

**Figure 1:**
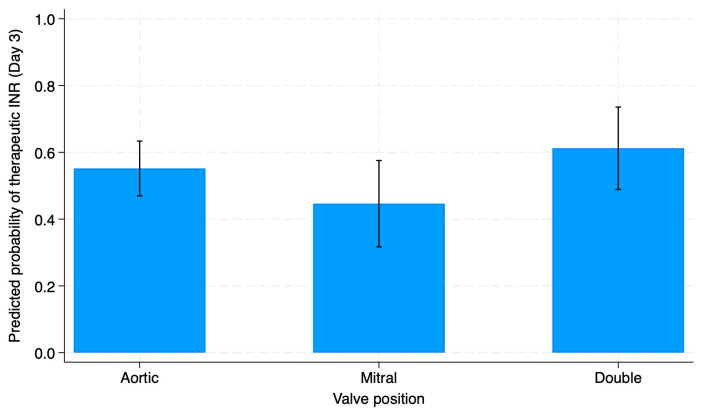
Adjusted predicted probability of achieving therapeutic INR on day 3 by valve position.

After adjusting for initial warfarin dose stratification, it was found that the predicted probability of achieving the therapeutic INR on day 3 varied by valve position; however, the difference was not statistically significant (*p* = 0.19). Patients with double valve replacement had the highest predicted probability to achieve the therapeutic INR by day 3 at approximately 0.61, while patients with mitral valve replacement had the lowest predicted probability, at around 0.45. However, the adjusted predicted probabilities differed numerically by valve position (Figure 1).

These findings indicate that valve position influences clinicians’ empirical warfarin dosing prescriptions as well as early INR target attainment.

### 3.4. Impact of Initial Warfarin Dose and Valve Type on INR Overshoot

Table 5 displays the proportion of patients with INR overshoot (INR ≥ 4) on postoperative day 3 across different valve positions and three initial warfarin dose groups: low (≤3 mg), moderate (4–5 mg), and high (>5 mg). INR overshoot on day 3 occurred in 14.29% of patients overall. Patients in the regular-dose group significantly (*p* = 0.028) showed the highest incidence of INR overshoot (18.90%), compared with 15.38% in the high-dose group and only 7.63% in the low-dose group. The highest rate of INR overshoot was also observed among mitral valve recipients (18.75%), followed by aortic (16.05%) and double valve recipients (4.55%). The association between valve position and INR overshoot was statistically significant (*p* = 0.033).

The results showed that the higher incidence of INR overshoot was independently associated with valve position and initial warfarin dosing.

### 3.5. Prediction of Initial Warfarin Dose Using LASSO Regression

To identify key clinical predictors of initial warfarin dosing, we employed a data-driven variable selection approach using LASSO regression, with EBIC-guiding variable selection. The model was trained on 296 observations and optimized using 10-fold cross-validation, selecting the optimal regularization parameter (λ = 0.042) based on the minimum prediction error (Table A1). Following variable selection, post-LASSO OLS regression was performed to obtain unbiased coefficient estimates and formulate the prediction equation. The final model included the following clinical predictors: age, valve position, heart failure status, sex, BMI, baseline INR, and albumin (see Table A2).

The final prediction model for the day 1 warfarin dose was

Predicted dose_day1 = 3.838 − 0.0226 × Age + 0.1653 × Valve Position − 0.4082 × Heart Failure − 0.4268 × Female + 0.1043 × BMI − 1.6547 × Baseline INR + 0.0187 × Albumin

This model explained approximately 27.9% of the variance in the day 1 warfarin dose (R^2^ = 0.2791) in the training dataset. Cross-validation yielded a cross-validated R^2^ of 0.201 and a mean-squared error (MSE) of 1.35, indicating moderate predictive accuracy.

**Figure 2:**
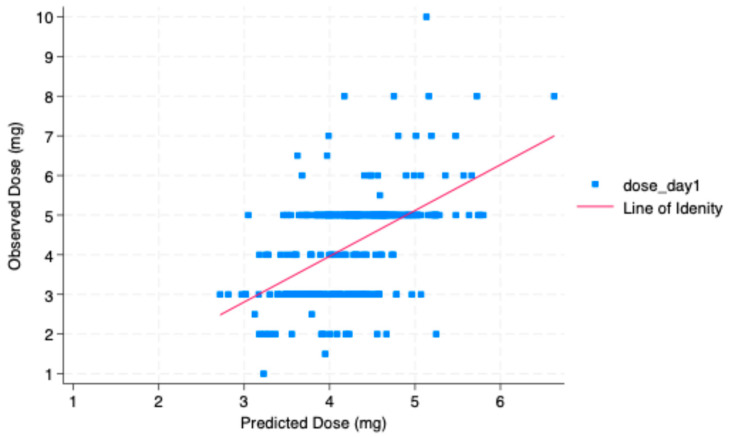
Relationship between predicted and observed warfarin dose on day 1.

Figure 2 illustrates the predicted and observed warfarin doses on day 1 with a Pearson correlation coefficient of r = 0.5209. The clustering of observed doses at standard increments (4 mg and 5 mg) reflects real-world empirical dosing. The variability around the identity line highlights the complexity of individual dose requirements and supports the model’s moderate accuracy in clinical dose prediction.

Overall, multiple demographic and clinical factors were found to be associated with early warfarin sensitivity and variability in initial dosing.

## 4. Discussion

Accumulating evidence has shown a high sensitivity to warfarin soon after HVR, which requires a lower initiating dosage compared with non-surgical patients [15,21,22]. Several clinical factors—such as age, body weight, concomitant medications, and comorbid conditions—can influence sensitivity to warfarin [7]. Despite this, warfarin therapy in these patient populations remains sub-optimally managed, with initiation protocols largely based on empirical dosing [4]. This study aimed to investigate postoperative changes that may affect warfarin sensitivity, with the goal of individualizing warfarin dosing and enhancing therapeutic management. In this study, we showed that patient sensitivity to warfarin after HVR is greatly impacted by the placement position of heart prostheses and, consequently, postoperative hemodynamic changes. Among patients who underwent HVR in different positions, we found that patients who underwent MVR were more likely to have heart failure and a decreased albumin level postoperatively. For this group of patients, we reported that the mean warfarin dose in the first 3 days was significantly lower than the mean warfarin doses received by other patients with different valve replacements in different positions. We found that clinicians empirically were more likely to choose lower initial doses for mitral valve recipients compared with aortic and double valve recipients, who commonly receive regular or higher doses. In the current study, the prevalence of heart failure and hypoalbuminemia was significantly higher among the mitral valve recipient group, which suggests a decreased dosage requirement in this patient population. Since warfarin is 99% bound to albumin, hypoalbuminemia significantly impacts its volume of distribution and half-life and, hence, enhances the warfarin response [11,15]. Additionally, Vorum et al. [23] reported a reduction in warfarin binding affinity in serum samples obtained within 3 days post-surgery. Supported by the current study results, the day 3 WDI3, a marker of early warfarin sensitivity, initially appeared to be higher in the MVR group compared with the other groups in the unadjusted analysis. To better understand this variability, we further investigated clinical factors associated with the WDI3 among patients undergoing HVR. After adjusting for relevant covariates, our multivariable analysis revealed that patients with mitral or double valve replacements exhibited significantly lower WDI3 values compared with those with aortic valve replacements, indicating reduced warfarin sensitivity in the early postoperative period. The decreased warfarin sensitivity in these subgroups possibly reflects the greater prothrombotic risk and the higher warfarin requirement needed by these patient groups to reach their therapeutic INR [9,10]

Additional factors influencing warfarin sensitivity include the baseline INR and gender. Female patients demonstrated significantly higher WDI3 values, consistent with established evidence that women are more sensitive to warfarin, likely due to differences in metabolism and protein binding [24,25]. Furthermore, the baseline INR also positively predicted the WDI3, suggesting that patients with a higher pre-treatment INR are more responsive to warfarin during early therapy [26]. Warfarin’s antithrombotic effects require a reduction in vitamin K-dependent clotting factors, particularly prothrombin (factor II), which has a relatively long half-life of 60 to 72 h [27]. Given that the antithrombotic effects of warfarin typically take 2 to 3 days to manifest after initiation [28], we selected day 3 as the landmark time point for analysis, aligning with the expected onset of warfarin’s anticoagulation effect.

During this study, only 50.65% of patients reached their therapeutic target by day 3. Our analysis showed that mitral valve recipients had a lower probability of achieving their therapeutic target, even after accounting for differences in their initial warfarin dosing. These results further support our early finding that warfarin sensitivity is reduced in mitral valve recipients, who may require higher doses to reach their desired therapeutic range. Furthermore, 14.29% of the patients in the current cohort showed an INR spike above 4 on day 3. Among these, MVR patients represented the largest proportion compared with the other groups. A previous study by Rahman et al., 2006 [21] showed that 25% of warfarin-treated patients post-MHV had an INR of ≥4.0 in the induction period. The higher frequency of patients exceeding the therapeutic range may be attributed to the initial dosing regimen used in that study, which involved administering 5 mg of warfarin on two consecutive days. Similarly, in our cohort, INR overshoot on day 4 was significantly more common among patients who received the standard initial doses (4–5 mg), with 32.6% experiencing an INR of >4.0, compared with 14.8% in the lower-dose group (≤3 mg) and 18.2% in the higher-dose group (>5 mg). Interestingly, although patients with MHV replacement received the lowest initial dose, they exhibited the highest risk of INR overshoot compared with patients with other valve types. These findings suggest that valve position should be considered when determining the initial warfarin dose to optimize anticoagulation management.

Considering the absence of a consensus guideline for initiating warfarin dosing in patients after HVR surgery, different studies have suggested using a loading dose of 5 mg for the majority of patients during the initial treatment period [4,21], while others have recommended applying lower doses for all patients [29]. Due to individualized differences in warfarin response that can be affected by many factors, it would be advantageous to apply a dosing algorithm to reduce the risk of high INRs during this period, thereby enhancing patient safety and reducing healthcare-associated costs [30]. In this study, we developed a clinical model to predict the initial warfarin dosing that could facilitate the choice of the appropriate dosing for each patient based on the clinical factors potentially affecting warfarin sensitivity. The clinical model we developed includes different clinical variables that were not previously captured by the previous clinically validated algorithm, including valve position, heart failure status, and albumin [30,31]. The day 1 dosing prediction model showed a moderate performance, explaining 27.9% of dose variance in the training dataset and yielding a cross-validated R^2^ of 0.201 with an MSE of 1.35. These findings suggest a moderate predictive accuracy and reinforce the model’s potential utility in guiding initial warfarin dosing decisions in clinical settings. However, a substantial portion of the variability remains unexplained, likely due to genetic factors and co-prescribed interacting medications that were not captured in the current model. To address these gaps, an ongoing clinical trial in Qatar is being conducted to investigate the clinical utility of a personalized approach that combines both genetic and clinical factors in managing patients receiving warfarin following HVR [32]. Supported by the current findings, this study presents an opportunity to enhance the existing clinically validated dosing algorithm for this patient population, with the goal of improving therapeutic outcomes. The results will contribute to the growing body of evidence on key factors that clinicians should consider when personalizing warfarin therapy.

## 5. Limitations

Several limitations should be acknowledged. This study’s retrospective, single-center design limits causal inference. The analysis was restricted to the early postoperative period (the first 3 days), which is clinically relevant but may not reflect later stabilization or long-term warfarin dose requirements. Detailed perioperative factors influencing warfarin response, including transfusion volumes, inflammatory status, vitamin K intake, and precise nutritional assessment, were not consistently available. Finally, pharmacogenetic data were not included, precluding evaluation of gene–drug interactions that may contribute to dosing variability. In a subsequent phase, we plan to incorporate genetic factors in a prospective cohort within the QPGx-CARES initiative, leveraging genotyping data to further improve warfarin dosing accuracy [32].

## 6. Conclusions

The results of this study demonstrate that several clinical factors are associated with increased sensitivity to initial warfarin dosing in patients following HVR surgery. These include the baseline INR, postoperative serum albumin concentration, valve position, heart failure status, and demographic factors such as age, gender, and BMI. Based on these findings, we recommend that patients presenting with any of these risk factors be carefully evaluated when determining their initial warfarin dose. To further enhance the personalization of warfarin therapy, these clinical factors should be considered alongside concomitant medications and genotyping data to improve dosing accuracy.

## Figures and Tables

**Table 1:** General preoperative patient characteristics.

Variable	Aortic Valve (n = 164)	Mitral Valve (n = 80)	Double Valve (n = 66)	*p*-Value
Age, years (mean ± SD)	47.70 ± 12.14	48.12 ± 10.77	49.75 ± 11.14	0.24
Female, n (%)	27 (16.46%)	31 (38.75%)	19 (28.78%)	0.001 *
BMI (mean ± SD)	28.89 ± 6.04	27.90 ± 5.83	28.04 ± 6.39	0.94
Diabetes, n (%)	72 (18.29%)	33 (41.35%)	29 (43.93%)	0.918
Heart failure, n (%)	30 (18.29%)	30 (37.5%)	22 (33.33%)	0.002 *
Albumin (mean ± SD)	38.04 ± 4.30	37.48 ± 4.10	39.16 ± 4.19	0.05 *
AST mean (IQR)	57.8 (34–62.5)	69.6 (52–82)	72.0 (51–89)	0.019 *
ALT mean (IQR)	22.3 (11–21)	19.7 (12–22.5)	22.3 (12–29)	0.77
PT (mean ± SD)	15.14 ± 8.99	14.77 ± 1.65	15.20 ± 5.14	0.92
Hemoglobin (mean ± SD)	9.42 ± 1.38	9.65 ± 1.16	9.19 ± 0.97	0.0812
WBC (mean ± SD)	10.05 ± 3.04	10.49 ± 3.12	10.74 ± 3.36	0.2665
Statin use, n (%)	86 (52.43%)	31 (38.75%)	30 (45.45%)	0.124

ALT—alanine aminotransferase, AST—aspartate aminotransferase, BMI—body mass index, IQR—interquartile range, and SD—standard deviation. * *p*-value < 0.05: significant.

**Table 2:** Warfarin dose and INR response in aortic, mitral and double valve groups.

Variable	Aortic Valve (n = 164)	Mitral Valve (n = 80)	Double Valve (n = 66)	*p*-Value
Mean warfarin dose (mg/day, D1–3)	4.35 ± 1.27	3.90 ± 1.18	4.58 ± 1.39	0.008 *
INR day 1 (mean ± SD)	1.54 ± 0.47	1.43 ± 0.29	1.47 ± 0.41	0.157
INR day 2 (mean ± SD)	2.41 ± 1.26	2.28 ± 1.55	1.85 ± 0.89	0.013 *
INR day 3 (mean ± SD)	2.71 ± 1.11	2.62 ± 1.36	2.22 ± 0.92	0.022 *
Baseline INR (mean ± SD)	1.27 ± 0.15	1.28 ± 0.14	1.27 ± 0.13	0.88
Day 3 INR ≥ 2.0 < 4, n (%)	91 (55.48%)	32 (41.25%)	33 (50%)	0.06
Day 3 INR ≥ 4, n (%)	26 (15.85%)	15 (18.75%)	3 (4.54%)	0.033 *
Warfarin dose index (WDI3)	0.25 ± 0.10	0.28 ± 0.14	0.22 ± 0.10	0.019 *

INR—international normalized ratio; * *p*-value < 0.05: significant.

**Table 3:** Multivariable linear regression of factors associated with day 3 warfarin dose index.

Variable	β Coefficient	Std. Error	*t*-Value	*p*-Value	95% CI (Lower–Upper)
Mitral/double valve (vs. aortic)	−0.023	0.009	−2.60	* 0.010	−0.0415825 to −0.0057093
Female (vs. male)	0.071	0.017	4.11	* 0.000	0.0373144 to 0.1058574
Baseline INR	0.141	0.05	2.73	* 0.007	0.039547 to 0.2438
Heart failure	0.0002424	0.0167467	0.01	0.988	−0.0327363 to 0.0332212
Albumin	0.0029251	0.0017151	1.71	0.089	−0.0004524 to 0.0063027
AST	0.0000544	0.0001522	0.36	0.721	−0.0002453 to 0.0003541

* *p*-value < 0.05: significant.

**Table 4:** Distribution of initial warfarin dose by valve position.

Valve Position	Low Dose (≤3 mg)	Regular Dose (4–5 mg)	High Dose (>5 mg)
Aortic (n = 164)	56 (34.15%)	95 (57.93%)	13 (7.93%)
Mitral (n = 80)	41 (51.25%)	34 (42.50%)	5 (6.25%)
Double (n = 66)	21 (31.82%)	36 (54.55%)	9 (13.64%)
Total	118 (38.06%)	165 (53.23%)	27 (8.71%)

Pearson chi-square (χ^2^) = 7.15; *p* = 0.028.

**Table 5:** Association of INR overshoot on day 3 with initial warfarin dose and valve position.

Variable	Category	INR < 4 (n)	INR ≥ 4 (n)	% Overshoot	*p*-Value
Initial warfarin dose	Low (≤3 mg)	109	9	7.63%	
	Regular (4–5 mg)	133	31	18.90%	
	High (>5 mg)	22	4	15.38%	* 0.028 ^1^
Valve position	Aortic	136	26	16.05%	
	Mitral	65	15	18.75%	
	Double	63	3	4.55%	* 0.033 ^2^
Total	308	264	44	14.29%	

^1^ Chi-square test for association between initial dose and INR overshoot; * *p* value < 0.05: significant. ^2^ Chi-square test for association between valve position and INR overshoot; * *p* value < 0.05: significant.

## Data Availability

The datasets generated and/or analyzed during the current study are available from the corresponding author upon reasonable request.

## Abbreviations

The following abbreviations were used in this manuscript:

AVR	Aortic valve replacement
EBIC	Extended Bayesian Information Criterion
HVR	Heart valve replacement
INR	International normalized ratio
LASSO	Least Absolute Shrinkage and Selection Operator
MSE	Mean-squared error
MVR	Mitral valve replacement
VHD	Valvular heart disease
WDI3	Warfarin dose index on day 3

## Appendix A

**Table A1:** Cross-validation results for LASSO linear regression model.

ID	Description	λ (Lambda)	Non-Zero Coefficients	CV R^2^	CV Prediction Error
1	First lambda	0.5184	0	−0.0026	1.6987
27	Lambda before *	0.0461	7	0.2013	1.3531
28	**Selected lambda**	**0.0420**	**7**	**0.2014**	**1.3531**
29	Lambda after	0.0383	7	0.2012	1.3533
34	Last lambda	0.0241	8	0.1997	1.3559

* Cross-validation was performed using 10 folds on 296 observations. The optimal lambda (bolded) was selected based on the minimum prediction error. CV R^2^: cross-validated coefficient of determination. Prediction error: mean-squared prediction error from cross-validation.

**Table A2:** LASSO-selected predictors of day 1 warfarin dose.

Variable	LASSO Coefficient	Post-Estimation OLS Coefficient
Age (years)	−0.0180	−0.0226
Valve position ^1^	+0.0808	+0.1653
Heart failure	−0.3194	−0.4082
Female (vs. male)	−0.2728	−0.4268
BMI (kg/m^2^)	+0.0923	+0.1043
Baseline INR	−1.2784	−1.6547
Albumin (g/dL)	+0.0115	+0.0187
Constant	+3.7474	+3.8339

^1^ Reference category for valve position = aortic valve. LASSO regression was used with EBIC-based penalty selection (λ = 26.88). Variables not selected by the model were excluded. The post-estimation OLS coefficients reflect refitted values using only the selected variables.
